# Attitude and level of COVID-19 vaccination and its determinants among patients with chronic disease visiting Debre Tabor Comprehensive Specialized Hospital, Northwest Ethiopia: A cross-sectional study

**DOI:** 10.1371/journal.pone.0278914

**Published:** 2022-12-09

**Authors:** Nega Dagnew Baye, Assefa Agegnehu Teshome, Atalo Agimas Ayenew, Anmut Tilahun Mulu, Endeshaw Chekol Abebe, Zelalem Tilahun Muche

**Affiliations:** Department of Biomedical Sciences, College of Health Sciences, Debre Tabor University, Debretabor, Ethiopia; Chinese Academy of Medical Sciences and Peking Union Medical College, CHINA

## Abstract

**Introduction:**

Coronavirus disease is a fatal viral disease caused by severe acute respiratory syndrome coronavirus 2. This study was aimed to assess the attitude, level of COVID-19 vaccine uptake, and its determinants among patients with chronic diseases visiting Debre Tabor Comprehensive Specialized Hospital, Northwest Ethiopia.

**Methods:**

An institutional-based cross-sectional study was conducted among 422 randomly selected patients with chronic disease visiting Debre Tabor Comprehensive Specialized Hospital from February 1 to March 30, 2022. Bivariable and multivariable binary logistic regression analyses were done to identify associations between dependent and independent variables.

**Results:**

Among all participants, only 29.6% of patients were vaccinated with any of the COVID-19 vaccines at least one dose. Age from 31 to 40 years (AOR = 6.26, 95% CI: 2.69–14.56), attended collage and above (AOR = 6.3, 95% CI: 1.37, 28.68), positive attitude towards COVID-19 vaccine (AOR = 9.07, 95% CI: 4.51–18.22), good knowledge (AOR = 7.63, 95% CI: 1.08–16.85), history of COVID-19 (AOR = 4.33, 95% CI: 1.85–10.17), family history of COVID-19 (AOR = 3.99, 95% CI = 1.89–8.48), ever been tested for COVID-19 (AOR = 0.33, 95% CI: 0.15–0.74) were determinant factors for COVID-19 vaccine uptake.

**Conclusion:**

COVID-19 vaccine uptake among patients with chronic disease was very low. The main reasons for not being vaccinated were doubts about vaccine efficacy, the vaccine may cause disease by itself, and fear of adverse effects. Therefore, different stakeholders should enforce vaccine uptake and awareness creation.

## Introduction

Coronavirus disease (COVID-19) is highly contagious viral infection caused by severe acute respiratory syndrome coronavirus 2 (SARS-CoV-2) [1]. SARS-CoV-2 belongs to the family of β-coronavirus which hinders pulmonary gas exchange and triggers cytokine storms. Intense inflammation, hypercoagulation, a drop in lymphocytic count, and an increase in neutrophilic count are observed in the second week after the onset of disease [2]. The clinical spectrum of COVID-19 is broad, ranging from mild flu-like symptoms to severe respiratory problems, organ dysfunction, and death. The symptoms of COVID-19 include fever, dry cough, sneezing, shortness of breath, and respiratory distress. Individuals with weak immunity, old age, diabetes, cardiovascular diseases, and other comorbidities are more susceptible to the attack of coronavirus infection [2, 3]. Since December 2019, COVID-19 has emerged as a major worldwide health threat, causing high rates of morbidity, mortality, serious economic and social impact [1]. The World Health Organization officially declared the spread of the COVID-19 virus as a global pandemic on March 11, 2020 and it is responsible for serious fatalities [4]. The virus has now spread all over the world, and the number of deaths continues to rise at an alarming rate [5]. As of April 21, 2022, it ravaged the world severely, with more than 505,035,185 confirmed cases and 6,210,719 deaths [6].

Among the different preventive measures to stop the spread of coronavirus, social distancing, vaccination, wearing gloves and face masks, and the use of sanitizer plays a vital role [2].

Vaccines are life-saving inventions that have helped to eradicate and control a wide range of infectious diseases. COVID-19 infection can be prevented through vaccination, especially in high-risk groups like healthcare professionals, the elderly, and people with chronic conditions [7]. Vaccination is safe, effective, and it is the most promising strategy to control the COVID-19 pandemic [8]. The vaccine has been shown to minimize infections even among those who have not been vaccinated, through the establishment of herd immunity, which occurs when a large percentage of the population is immunized [9]. Countries around the world may need to make a greater effort in vaccination policies to control the pandemic and resume normal living [10].

Since the end of 2020, several COVID-19 vaccines have been officially approved with emergency use authorization and provided to the population. This includes adenoviral vector (Ad26/Ad5) vaccines of Gamaleya’s Sputnik V (Gam-COVID-Vac) in Russia, Pfizer-BioNTech in the US, Moderna COVID-19 mRNA vaccines in EU, AstraZeneca (AZ) in UK and EU, inactivated Bharat Biotech BBV152 COVAXIN in India, Johnson and Johnson (J&J)/Janssen in the US, Anhui-Zhifei recombinant protein vaccine, Sinopharm, Sinovac and CanSino (adenovirus 5 vector) in China [11]. The common side effects of COVID-19 vaccine are pain at the injection site, fever, headache, tiredness, muscle pain, joint pain, chest pain, dizziness, and chills [12, 13].

Even though a great effort is being made in vaccination, vaccine hesitancy and resistance are continued as a major obstacles to achieve a successful vaccination campaign around the world, with a large section of the population in many countries refusing to be vaccinated against COVID-19 [14, 15]. Questions about a vaccine’s safety and a general lack of confidence, as well as concerns about the vaccine’s efficacy, were the most common reasons for hesitancy [7, 16, 17]. Multiple beliefs and misconceptions among various population groupings can influence acceptance of the COVID-19 vaccination [18]. In low-resource settings, the refusal to take this vaccine is more pronounced [19]. Since March 7, 2021, the Ethiopian Ministry of Health has officially initiated COVID-19 immunization for prioritized populations such as health professionals, the elderly, and patients with chronic diseases above the age of 55. In addition, on November 16, 2021, the Ministry initiated a COVID-19 vaccination program with the goal of vaccinating everyone aged 12 and above. For the campaign, it has distributed over 6.2 million doses of COVID-19 vaccine (Sinopharm, AstraZeneca, Johnson, and Pfizer Biontch). Only the Pfizer Biontch vaccine was provided to individuals aged 12 to 18 years, while the other vaccines were given to those aged 18 years and above across the country. Jansen is given in a single dose and all others are given in two doses where the second dose is administered 3 to 4 weeks after the first dose, except for AstraZeneca where the second dose is given after 6–12 weeks of the first dose [10].

Therefore, understanding the existing level of vaccination and identifying common barriers and facilitators of uptake of the COVID-19 vaccine is crucial for increasing vaccination coverage and reducing COVID-19 cases, hospitalizations, and fatalities.

To our knowledge, there is no prior study on the level of COVID-19 vaccine uptake and determinant factors among patients with chronic disease in the study area. Therefore, this study was aimed to assess the attitude, level of COVID-19 vaccine uptake, and its determinants among patients with chronic diseases visiting Debre Tabor Comprehensive Specialized Hospital, Northwest Ethiopia.

## Materials and methods

### Study design and setting

An institutional-based cross-sectional study was conducted among 422 patients with chronic disease visiting Debre Tabor Comprehensive Specialized Hospital from February 1 to March 30, 2022. The hospital is located in Debre Tabor town which is the zonal center of South Gondar Zone, Amhara region, Northwest Ethiopia. The town is located 667 km from Addis Ababa and 98 km from Bahir Dar. The hospital serves around 2.2 million population across the region and provides a broad range of medical services for all age groups.

### Participants

The source populations were all patients with chronic diseases who had visited Debre Tabor Comprehensive Specialized Hospital. While all patients with chronic diseases who had visited the hospital during the data collection time were study populations.

All patients with chronic diseases who are aged above 18 years and volunteered to participate in the study during the data collection period were included in the study. However, those who did not have the willingness to participate and were critically ill (unable to respond) were excluded.

### Study variables

COVID-19 vaccine uptake (yes/no response) was the outcome variable. The independent variables included Sociodemographic (age, sex, ethnicity, religion, occupation, educational status, marital status, and income), health status-related (history of contact with confirmed COVID-19 patients, family history of COVID-19, ever been tested for COVID-19, history of COVID-19 infection, type of chronic diseases), knowledge and attitude towards COVID-19 vaccine.

### Sample size determination and sampling technique

The sample size was determined using a single population proportion formula by considering a 95% confidence interval (CI), p = 50% (proportion of vaccination among chronically diseased patients), 5% margin of error, and a 10% non-response rate. The final sample size was 422. Based on the eligibility criteria, a systematic random sampling technique was employed until the necessary samples were obtained.

### Operational definitions

**COVID-19 vaccine uptake:** is the number of people who have received a specified vaccine dose (at least one dose of a COVID-19 vaccine) during the study period. It was assessed by the closed-ended question “Have you been vaccinated with any of the COVID-19 vaccines at least once currently?” and the responses were “Yes” and “No [20].

**Attitude to COVID-19 vaccine:** The attitude of the respondents was determined based on 8 attitude assessment questions. Respondents who scored greater than or equal to the mean score of attitude assessment questions were considered to have a positive attitude, while those who scored less than the mean score were considered as having a negative attitude towards the COVID-19 vaccine [21, 22].

**Knowledge on COVID-19 vaccine:** was assessed by six knowledge assessment questions. Participants who scored greater than or equal to the mean score of knowledge assessment questions were considered as having good knowledge, while those who scored less than the mean score were considered as having poor knowledge towards the COVID-19 vaccine [21, 22].

### Data collection and quality assurance

The data were collected by five nurse professionals through a face-to-face interview method using the pretested structured questionnaire, which was adapted after reviewing relevant literatures. The questionnaire was prepared first in English and translated into Amharic and then translated back to English to ensure the accuracy of the translated version. The questionnaires included socio-demographic, health status-related, COVID-19 vaccine uptake, and knowledge and attitude towards COVID-19 vaccine-related variables.

Before the actual data collection, a pretest was done on 5% of study participants at Ebinat Primary Hospital. Three days of training were also given for data collectors. Daily supervision was done during the data collection period.

### Statistical analysis

After the collected data were checked for completeness, clarity, and accuracy, it was entered into EPI Info version 7.7.1 and then exported to Statistical Package for Social Sciences (SPSS) version 26 for analysis. The characteristics of the study participants were analyzed using descriptive statistics such as frequency and percentage and presented in tables and figure. Both bivariate and multivariate logistic regression analyses were used to explore the association between the dependent and the outcome variable. In the bivariable analysis, variables with p-values of less than 0.2 were entered into multivariable analysis to analyze the association between the outcome variable and predictor variables. Then in multivariable analysis, variables with p-values of less than 0.05 were taken as determinant factors significantly associated with COVID-19 vaccine uptake.

### Ethical considerations

The study was conducted after ethical approval was obtained from the ethical review committee of the College of Health Sciences, Debre Tabor University. Written informed consent which was approved by the ethical review committee was obtained from each participant after the purpose and procedure of the study was well explained and their willingness to participate in the study or not was asked. The participants were assured that their responses will remain secured and confidential.

## Results

### Socio-demographic characteristics of participants

A total of 422 chronically diseased patients of different types were included in the study, with a response rate of 100%. The mean age of participants was 43.2 years (SD-13.7 years) with 29.6% aged between 41 to 50 years. Nearly half (227, 53.8%) of the respondents were males and 109 (25.8%) were government employees. Majority of participants belonged to Amhara, 410 (97.2%) by ethnicity and Orthodox Christian, 358 (84.8%) by religion. Around 173 (41%) participants attended college or above (**Table 1**).

**Table 1:** Socio-demographic characteristics of patients with chronic disease visiting Debre Tabor Comprehensive Specialized Hospital, Northwest Ethiopia, 2022.

Variables		Frequency (%)
Age	18–30	90 (21.3%)
	31–40	104 (24.6%)
	41–50	125 (29.6%)
	50 and above	103 (24.4%)
Sex	Male	227 (53.8%)
	Female	195 (46.2%)
Marital status	Married	263 (62.3%)
	Single	91 (21.6%)
	Widowed	43 (10.2%)
	Divorced	25 (5.9%)
Educational status	No formal education	46 (10.9%)
	Primary education	94 (22.3%)
	Secondary education	109 (25.8%)
	Collage and above	173 (41%)
Occupation	Housewife	55 (13%)
	Farmer	28 (6.6%)
	Private employ	70 (16.6%)
	Daily laborer	39 (9.2%)
	Merchant	85 (20.1%)
	Gov’t employs	109 (25.8%)
	Other	36 (8.5%)
Ethnicity	Amhara	410 (97.2%)
	Oromo	9 (2.1%)
	Tigrai	3 (0.7%)
Religion	Orthodox	358 (84.8%)
	Catholic	9 (2.1%)
	Protestant	37 (8.8%)
	Muslim	18 (4.3%)
Family size	<5	308 (73%)
	≥5	114 (27%)
Monthly income	<5000ETB	112 (26.5%)
	5000-9999ETB	179 (42.4%)
	10,000-1499ETB	98 (23.2%)
	≥15,000ETB	33 (7.8%)
Residence	Rural	133 (31.5%)
	Urban	289 (68.5%)

### Health-related characteristics of participants

Nearly two-thirds of study participants (288, 68.3%) were five years or above since being diagnosed with the disease. Hypertension (33.4%) was the most commonly reported chronic disease followed by diabetes mellitus (19.9%). About 83 (19.7%) patients had a history of COVID-19 infection and 86 (20.4%) of participants had a family history of COVID-19 infection. Around 138 (32.7%) participants had history of contact with confirmed COVID-19 patients. Of 119 (28.2%) respondents who had been tested for COVID-19, about 43.7% of them had a positive test result. More than half (297, 70.4%) thought that the disease will make them more vulnerable to COVID-19 than non-diseased individuals (**Table 2**).

**Table 2:** Health status-related characteristics of patients with chronic disease visiting Debre Tabor Comprehensive Specialized Hospital, Northwest Ethiopia, 2022.

Variables		Frequency	Percentage
Duration since diagnosed with the disease	<5 years	134	31.8%
	5–10 years	200	47.4%
	>10 years	88	20.9%
Type of chronic disease	Hypertension	141	33.4%
	Diabetes mellitus	84	19.9%
	Heart disease	37	8.8%
	HIV/AIDS	41	9.7%
	Renal disease	32	7.6%
	Respiratory disease	58	13.7%
	Other*	29	6.9%
History of COVID-19 infection	Yes	83	19.7%
	No	339	80.3%
Family History of COVID-19 infection	Yes	86	20.4%
	No	336	67.3%
History of contact with confirmed COVID-19 case	Yes	138	32.7%
	No	284	67.6%
Tested for COVID-19 infection	Yes	119	28.2%
	No	303	71.8%
If tested, the test result	Positive	52	43.7%
	Negative	67	56.3%
The disease makes them more vulnerable to COVID-19	Yes	297	70.4%
	No	125	29.6%

### Knowledge towards COVID-19 vaccine

Less than half 168 (39.8%) of participants had good knowledge towards COVID-19 vaccine. Majority 341 (80.9%) of the respondents correctly replied that COVID-19 vaccines are currently given in Ethiopia. About 172 (40.7%) study participants knew that vaccines are important to prevent COVID-19. Furthermore, 166 (39.3%) participants knew that COVID-19 vaccines are safe for patients with chronic illnesses (**Table 3**).

**Table 3:** Knowledge towards COVID-19 vaccine among patients with chronic disease visiting Debre Tabor Comprehensive Specialized Hospital, Northwest Ethiopia, 2022.

*Variables*	*Response*	*Frequency (%)*
*Do you think that COVID-19 vaccine is currently given in Ethiopia?*	Yes	341 (80.9)
	No	81 (19.1)
*Do you think vaccines are important to prevent COVID-19?*	Yes	172 (40.7)
	No	250 (59.3)
*Do you think that the COVID-19 vaccine has side effects or risks?*	Yes	196 (46.4)
	No	226 (53.6)
*Do you think that COVID-19 vaccines are effective?*	Yes	164 (38.8)
	No	258 (61.2)
*Do you think patients with chronic disease should be given priority for the vaccine?*	Yes	248 (58.8)
	No	274 (41.2)
*Do you think the COVID-19 vaccine is safe in patients with chronic illness?*	Yes	166 (39.3)
	No	256 (60.7)

### Attitude towards COVID-19 vaccine

Less than half 172 (40.8%) of the respondents had a positive attitude towards the COVID-19 vaccine. Only 36.5% of participants agreed that COVID-19 vaccines are safe, and 39.3% of respondents agreed that COVID-19 vaccination is mandatory for all populations. Similarly, about half (51.7%) of the respondents agreed that the vaccine is accessible to all. Nearly half of the respondents (47.4%) agreed that the vaccine could reduce the spread of the virus in the community (**Table 4**).

**Table 4:** Attitude towards COVID-19 vaccine among patients with chronic disease visiting in Debre Tabor Comprehensive Specialized Hospital, Northwest Ethiopia, 2022.

*Variables*	*Response*	*Frequency (%)*
*COVID-19 can be prevented by the vaccine*.	Agree	247 (58.5)
	Neutral	57 (13.5)
	Disagree	118 (28)
*The current COVID-19 vaccine is effective*.	Agree	162 (38.4)
	Neutral	107 (25.4)
	Disagree	153 (36.3)
*COVID-19 vaccine will not be effective in patients with chronic disease*.	Agree	115 (27.3)
	Neutral	189 (44.7)
	Disagree	118 (28)
*COVID-19 vaccine is safe in patients with chronic illness*.	Agree	154 (36.5)
	Neutral	115 (27.3)
	Disagree	153 (36.3)
*The information given by official media on COVID-19 vaccine is trustable*.	Agree	209 (49.5)
	Neutral	68 (16.1)
	Disagree	142 (33.6)
*COVID-19 vaccine can reduce the spread of the virus in the community*.	Agree	200 (47.4)
	Neutral	111 (26.3)
	Disagree	111 (26.3)
*It is not possible to reduce COVID-19 without vaccination*.	Agree	169 (39.8)
	Neutral	78 (18.5)
	Disagree	176 (41.7)
*COVID-19 vaccine should be mandatory for all populations*.	Agree	166 (39.3)
	Neutral	99 (23.5)
	Disagree	157 (37.2)

### COVID-19 vaccine uptake

Of all study participants, 125 (29.6%) were vaccinated with any of the COVID-19 vaccines at least one dose. Of those participants who took the vaccine (n = 125), 39 (31.2%) had received the vaccine in the first round. Majority of the respondents had no symptoms after vaccination. However, 40% of respondents had symptoms after vaccination: the main symptoms reported were fever (56.3%), chills (10%), and headache (18%).

### Reasons for not being vaccinated COVID-19 vaccine

**Fig 1:**
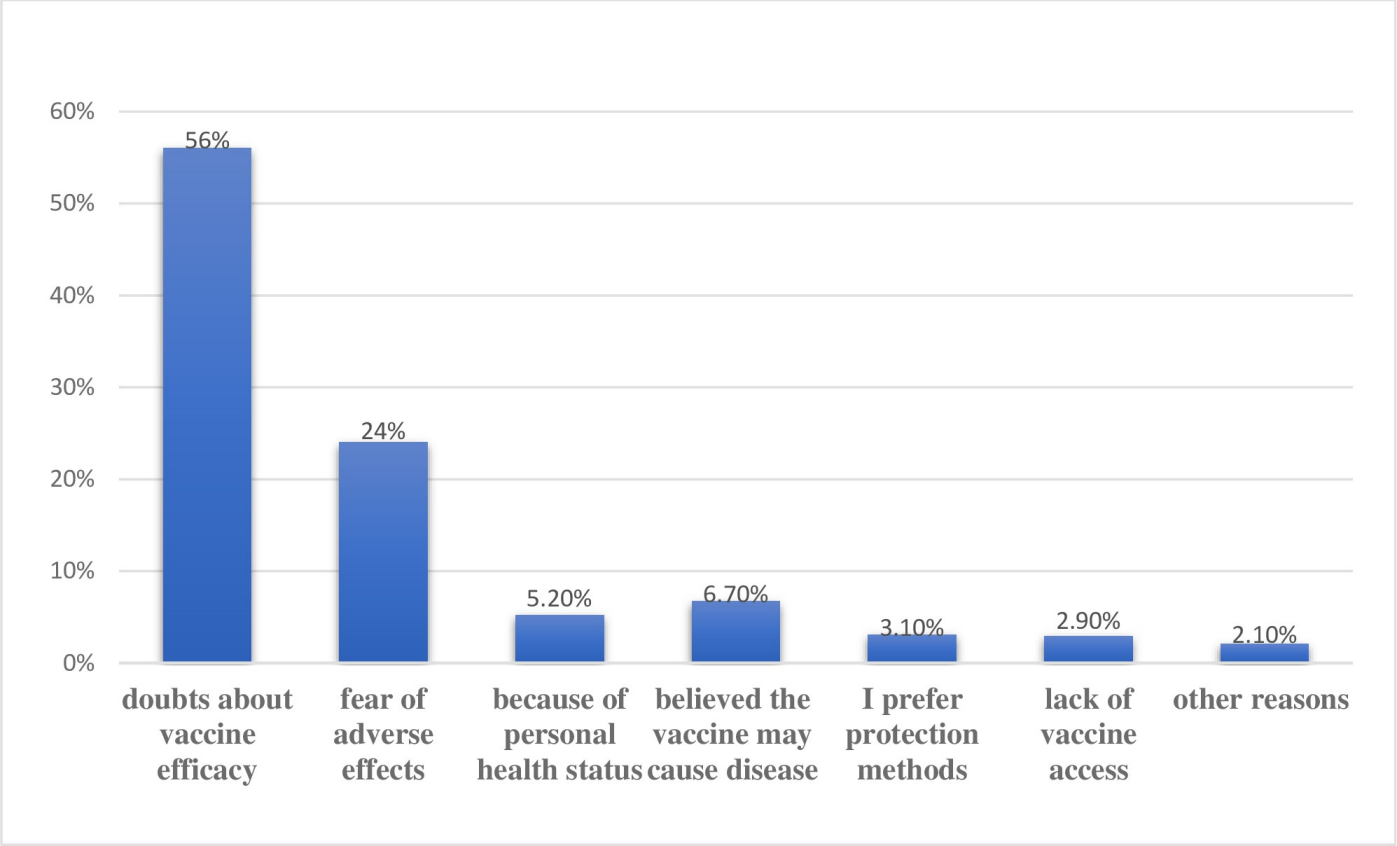
Respondents’ main reason for not being vaccinated in patients with chronic disease, 2022.

The main reasons for not receiving the vaccine were 166 (56%) doubts about vaccine efficacy, 72 (24%) fear of adverse effects, 20 (6.7%) believed the vaccine may cause disease by itself, 16 (5.2%) unable to withstand the vaccine because of an underlying chronic disease, and 9 (3.1%) prefer other non-vaccine preventive measures (Fig 1).

### Determinant factors of COVID-19 vaccine uptake

In multivariable binary logistic regression analysis, variables such as age, educational status, history of COVID-19 infection, family history of COVID-19, ever been tested for COVID-19, knowledge, and attitude towards COVID-19 vaccine were statistically associated with COVID-19 vaccine uptake. However, some variables such as religion, ethnicity, marital status and residence, types of chronic disease, income, and family sizes were excluded because their p-value was >0.2 on binary logistic regression analysis.

Patients aged from 31 to 40 years were 6.3 times more likely to uptake COVID-19 vaccine (AOR = 6.26, 95% CI: 2.69–14.56) and younger age (18–30 years) patients were 4.47 times (AOR = 4.47, 95% CI: 1.74–11.5) more likely to get vaccinated than older age groups. Patients who had a positive attitude towards the COVID-19 vaccine were 9 times more likely to uptake the COVID-19 vaccine (AOR = 9.07, 95% CI: 4.51–18.22) than those who had a negative attitude. The likelihood of receiving the COVID-19 vaccine was 7.63 times (AOR = 7.63, 95% CI: 1.08–16.85) higher in those patients with chronic diseases who had good knowledge about the vaccine than in those who had poor knowledge. Those who had a history of COVID-19 infection were 4.33 times (AOR = 4.33, 95% CI: 1.85–10.17) more likely to uptake the COVID-19 vaccine than those who had no history of COVID-19 infection. Participants who had a family history of COVID-19 were 4 times more likely to uptake COVID-19 vaccine (AOR = 3.99, 95% CI: 1.89–8.48) compared to those who had no family history of COVID-19. The probability of COVID-19 vaccine uptake by those who had been tested for COVID-19 was reduced by 32.9% (AOR = 0.329, 95% CI: 0.15–0.74) compared to those who had never been tested for COVID-19 (Table 5).

**Table 5:** Determinant factors of COVID-19 vaccine uptake among patients with chronic disease visiting Debre Tabor Comprehensive Specialized Hospital, Northwest Ethiopia, 2022.

Variables	COVID-19 vaccine uptake		COR (95%CI)	AOR (95%CI)	P-value
	Yes (%)	No (%)			
Age					
18–30	30 (33.3%)	60 (66.7%)	3.18 (1.56–6.49)	4.47 (1.74–11.50)	0.00*
31–40	44 (42.3%)	60 (57.7%)	4.66 (2.35–9.25)	6.26 (2.69–14.56)	0.02*
41–50	37 (29.6%)	88 (70.4%)	2.67 (1.35–5.29)	3.87 (1.59–9.43)	0.003*
50 and above	14 (13.6%)	89 (86.4%)	1	1	
Sex					
Male	25 (25.1%)	170 (74.9%)	1	1	
Female	68 (34.9%)	127 (65.1%)	1.6 (1.04–2.44)	1.56 (0.81–7.86)	0.23
Educational status					
No formal education	5 (10.9%)	41 (89.1%)	1	1	
Primary education	32 (34%)	62 (66%)	4.23 (1.52–11.76)	4.84 (1.29–18.04)	0.01*
Secondary education	34 (31.2%)	75 (68.8%)	3.71 (1.35–10.23)	3.11 (1.06–11.83)	0.04*
Collage and above	54 (31.2%)	119 (68.8%)	3.72 (1.39–9.94)	2.77 (1.09–10.01)	0.01*
Duration since diagnosed with the disease					
<5 years	46 (34.3%)	88 (65.7%)	1	1	
5–10 years	51 (25.5%)	149 (74.5%)	0.65 (0.41–1.06)	0.58 (0.30–1.15)	0.12
>10 years	28 (31.8%)	60 (68.2%)	0.89 (0.0.5–1.58)	0.51 (0.21–1.21)	0.13
Attitude towards COVID-19 vaccine					
Positive	99 (57.6%)	73 (42.4%)	7.97 (4.61–13.76)	9.07 (4.51–18.22)	0.000**
Negative	26 (10.4%)	224 (89.6%)	1	1	
Knowledge towards COVID-19 vaccine					
Good	102 (60.7%)	66 (39.3%)	7.04 (1.68–16.85)	7.63 (1.08–16.85)	0.000**
Poor	23 (9.1%)	231 (90.9%)	1	1	
History of COVID-19 infection					
Yes	47 (56.6%)	36 (43.4%)	4.37 (2.64–2.43)	4.33 (1.85–10.17)	0.001*
No	78 (23%)	261 (77%)	1	1	
Family History of COVID-19 infection					
Yes	50 (58.1%)	36 (41.9%)	4.83 (2.93–7.96)	3.99 (1.89–8.48)	0.0001**
No	75 (22.3%)	261 (77.7%)	1	1	
Ever been test for COVID-19					
Yes	70 (58.8%)	49 (41.2%)	2.09 (1.33–3.27)	0.33 (0.15–0.74)	0.007*
No	76 (25.1%)	227 (74.9%)	1	1	

*P-value<0.05,

**P-value<0.001, COR- Crude odds ratio, AOR- Adjusted odds ratio, CI-Confidence interval

## Discussion

The COVID-19 pandemic has become a major public health concern, resulting in high morbidity and mortality. The likelihood of acquiring hazardous COVID-19 symptoms is higher in high-risk population groups, such as people with chronic illnesses [23, 24]. Hence, priority access to the COVID-19 vaccine should be offered to these higher-risk populations.

About 83 (19.7%) of study participants have been infected with COVID-19, which is consistent with a study conducted among cancer patients in Addis Ababa [25]. In this study, 125 (29.6%) patients with chronic disease were vaccinated for COVID-19 at least once. This finding was significantly higher than the result reported from Addis Ababa (14.5%) [25]. However, it was lower than the study findings in China (84.22%) [26], France (69%) [27], Ontario, Canada (81.6%) [28], US (57%) [29], Harar region, Ethiopia (39.4%) [30], and Pennsylvania (59.3%) [31]. This discrepancy could be attributed to the study subjects being the general public, differences in awareness among the population groups, and easier access to information.

In this study, less than half (39.8%) of the patients had good knowledge towards the COVID-19 vaccine which was inline with the finding in Ethiopia (40.8%) [22]. However, it was lower than the finding reported from Dessie Comprehensive Specialized Hospital (62.7%) [21]. This low level of knowledge towards the vaccine may be attributed to the change in the target population, educational status, and use of different sources of information towards COVID-19 vaccines. About 40.8% of the participants had a positive attitude towards the vaccine which was higher than the finding in Ethiopia (24.2%) [22] but lower than the findings in Dessie Comprehensive Specialized Hospital (71.6%) [21] and China (70.07%) [26]. The possible reason for this variation may be due to the poor risk perception towards COVID-19.

Age, educational status, history of COVID-19 infection, ever been tested for COVID-19, family history of COVID-19, knowledge, and attitude toward COVID-19 vaccine were all found to be determinants of COVID-19 vaccine uptake in this study. COVID-19 vaccine uptake was also observed to be higher among younger and middle-aged patients with chronic disease (18–30, 31–40, and 41–50) compared to older patients with chronic disease (50 and above years). This is consistent with a study conducted in Addis Ababa [25]. However, it is incontrary to a study in US, which found that COVID-19 vaccination was highest in older adults [29].

In our study, patients who attended college and above were 2.77 times more likely to be vaccinated than those with no education. This is inconsistent with a study in the Harar region, Ethiopia [30] where people who had no schooling were 2.5 times more likely to be vaccinated than people who had attended above-secondary school. This could be due to the fact that educated people are more likely to accept the recommended vaccine.

Respondents with a history of COVID-19 infection were three times more likely than those without a history of COVID-19 illness to take the COVID-19 vaccine. In contrast, respondents with a history of COVID-19 infection were less likely to be vaccinated in research conducted in France [27].

Furthermore, participants with a good attitude toward a COVID-19 vaccine were 9 times more likely to get vaccinated, which was consistent with the data from Addis Ababa [25] and Dessie [21], which found that patients with a positive attitude were more likely to accept the vaccine. This was also true in Ethiopia [22] and China [26] where respondents who had a positive attitude towards the COVID-19 vaccine were more likely to uptake it. The possible reason might be a positive attitude toward COVID-19 vaccine may avoid misinformation regarding the vaccine and outweigh its importance and then encourage people to be vaccinated.

Doubts about vaccine efficacy (56%) and fear of adverse effects (24%) were the most common reasons for not being vaccinated, while in China the unvaccinated population (23.91%) is primarily due to personal health status [26]. A consistent finding was obtained from a study conducted among cancer patients in Addis Ababa where 84.4% of them had concerns about the safety and side effects of the vaccine [25].

The study has the following limitations: firstly, it was a cross-sectional study in which the identification of causal factors may be very difficult, and secondly, it is hard to follow up the future behaviors of those who have not been vaccinated.

## Conclusion

The study revealed that COVID-19 vaccine uptake (29.6%) among patients with chronic illness was still low. Age, educational status, history of COVID-19 infection, ever been tested for COVID-19, family history of COVID-19, knowledge and attitude towards COVID-19 vaccine were all determinant factors that influence COVID-19 vaccine uptake among patients with chronic disease in Ethiopia. Doubts about vaccine efficacy, the vaccine may cause disease by itself, and fear of adverse effects were the primary reasons for not being vaccinated.

As a result, decision-makers and health managers should update and devise strategies to ensure COVID-19 vaccination uptake in conjunction with various stakeholders. The South Gondar Zone Health Bureau should continue to raise awareness about the safety, importance, and efficacy of the COVID-19 vaccine.
